# Inequalities in Exposure to Firearm Violence by Race, Sex, and Birth Cohort From Childhood to Age 40 Years, 1995-2021

**DOI:** 10.1001/jamanetworkopen.2023.12465

**Published:** 2023-05-09

**Authors:** Charles C. Lanfear, Rebecca Bucci, David S. Kirk, Robert J. Sampson

**Affiliations:** 1 Institute of Criminology, University of Cambridge, Cambridge, United Kingdom; 2 Department of Sociology, Harvard University, Cambridge, Massachusetts; 3 Department of Sociology and Nuffield College, University of Oxford, Oxford, United Kingdom

## Abstract

**Importance:**

The past quarter-century has seen both sharp declines and increases in firearm violence in the United States. Yet, little is known about the age of first exposure to firearm violence and how it may differ by race, sex, and cohort.

**Objective:**

To examine race, sex, and cohort differences in exposure to firearm violence in a representative longitudinal study of children who grew up in periods with varying rates of firearm violence in the United States and to examine spatial proximity to firearm violence in adulthood.

**Design, Setting, and Participants:**

This population-based representative cohort study included multiple cohorts of children followed-up from 1995 through 2021 in the Project on Human Development in Chicago Neighborhoods (PHDCN). Participants included Black, Hispanic, and White respondents from 4 age cohorts of Chicago, Illinois, residents, with modal birth years of 1981, 1984, 1987, and 1996. Data analyses were conducted from May 2022 to March 2023.

**Main Outcomes and Measures:**

Firearm violence exposure, including age when first shot, age when first saw someone shot, and past-year frequency of fatal and nonfatal shootings within 250 m of residence.

**Results:**

There were 2418 participants in wave 1 (in the mid-1990s), and they were evenly split by sex, with 1209 males (50.00%) and 1209 females (50.00%). There were 890 Black respondents, 1146 Hispanic respondents, and 382 White respondents. Male respondents were much more likely than female respondents to have been shot (adjusted hazard ratio [aHR], 4.23; 95% CI, 2.28-7.84), but only moderately more likely to have seen someone shot (aHR, 1.48; 95% CI, 1.27-1.72). Compared with White individuals, Black individuals experienced higher rates of all 3 forms of exposure (been shot: aHR, 3.05; 95% CI, 1.22-7.60; seen someone shot: aHR, 4.69; 95% CI, 3.41-6.46; nearby shootings: adjusted incidence rate ratio [aIRR], 12.40; 95% CI, 6.88-22.35), and Hispanic respondents experienced higher rates of 2 forms of violence exposure (seen someone shot: aHR, 2.59; 95% CI, 1.85-3.62; nearby shootings: aIRR, 3.77; 95% CI, 2.08-6.84). Respondents born in the mid-1990s who grew up amidst large declines in homicide but reached adulthood during city and national spikes in firearm violence in 2016 were less likely to have seen someone shot than those born in the early 1980s who grew up during the peak of homicide in the early 1990s (aHR, 0.49; 95% CI, 0.35-0.69). However, the likelihood of having been shot did not significantly differ between these cohorts (aHR, 0.81; 95% CI, 0.40-1.63).

**Conclusions and Relevance:**

In this longitudinal multicohort study of exposure to firearm violence, there were stark differences by race and sex, yet the extent of exposure to violence was not simply the product of these characteristics. These findings on cohort differences suggest changing societal conditions were key factors associated with whether and at what life stage individuals from all race and sex groups were exposed to firearm violence.

## Key Points

**Question:**

Did the likelihood of being shot, seeing someone shot, or living near firearm violence vary by race, sex, and birth cohort over the life course during the past 3 decades?

**Findings:**

This cohort study found that the likelihood of exposure to all forms of firearm violence varied significantly and persistently over the life course by race. Sex differences were greatest for being shot compared to witnessing or proximity to firearm violence, and cohort differences were most pronounced for witnessing violence.

**Meaning:**

These finding suggest that understanding who gets exposed to firearm violence and when requires simultaneous attention to inequality by race, sex, and cohort.

## Teaser

This cohort study examines race, sex, and cohort differences in exposure to firearm violence in a representative longitudinal study of children who grew up in a large US city during periods with varying rates of firearm violence.

## Introduction

Nearly 21 000 individuals in the US were killed by gun violence in 2021, a rate of 6.3 deaths per 100 000 population.^1^ A rate of this magnitude has not been seen since 1994, near the late–20th-century peak in firearm violence.^2^ After that, an unexpectedly steady and sizable multidecade decline in violence took place, with the national gun homicide rate decreasing to 3.5 deaths per 100 000 population by 2014. Taking experts by surprise, homicides then began increasing before spiking in 2016 and again in 2020.^3^ Firearm deaths have now surpassed motor vehicle crashes as the leading cause of death for US children, with Black children, boys, and children from disadvantaged neighborhoods in cities like Chicago, Illinois, disproportionately impacted.^4,5,6,7^

The extreme fluctuation in lethal violence in the US over the past 3 decades means that successive cohorts of children growing up and reaching adulthood in these times experienced much different social worlds, what has been called the “birth lottery of history.”^8^ Even if they are from the same socioeconomic status, some adults today were raised during an era of relative calm, whereas others, sometimes just a handful of years older or younger, reached their formative years of adolescence and early adulthood during epidemic levels of firearm violence.

Despite increasing attention to the burden of firearms on health and mortality, exposure to firearm violence over the life course is not well understood, particularly exposure to nonfatal firearm violence.^9^ Much of the focus has been on national estimates of homicides and suicides. Other studies have sought to examine the causes of aggregate changes in violence over recent decades.^10,11^ While important, such studies do not reveal when in the life course exposure occurs and whether dynamic patterns differ by race, sex, and birth cohort. Long-term longitudinal studies of exposure to firearm violence are relatively rare, especially those that document how both witnessing violence and personally experiencing violence unfold over the life course and differ by race and sex.^12,13^

Rarer still are studies that examine different cohorts who reached late adolescence and transitioned to adulthood during times of historic highs and lows in violence over the last quarter-century. Without studies of multiple cohorts, it is challenging to pinpoint whether and how broad societal changes, such as the 1990s and 2000s crime decline, the steady weakening of gun laws, or the COVID-19 pandemic, might have influenced exposure to firearm violence by age.

In this study, we leverage a unique longitudinal design to examine exposure to firearm violence from childhood through midadulthood by comparing the likelihood of being shot as well as seeing someone shot for multiple cohorts of children from Chicago, separated in age by approximately 15 years. Although followed over the same period, 1995 to 2021, the children from these cohorts reached key developmental periods in the life course during much different societal contexts. We highlight cohort inequalities in exposure to firearm violence, as well as disparities by race and sex. Finally, we examine race, sex, and birth cohort differences in spatial proximity to firearm violence in the present decade.

## Methods

This cohort study was approved by the Harvard University institutional review board. Adult respondents provided written or verbal informed consent to participate in the study, and parents consented for child respondents. This study adhered to the Strengthening the Reporting of Observational Studies in Epidemiology (STROBE) reporting guideline for cohort studies.

### Sample and Procedures

The Project in Human Development in Chicago Neighborhoods (PHDCN) began in the mid-1990s as a representative sample of 6207 age-eligible children drawn from a screening of more than 35 000 households in a stratified representative sample of 80 of Chicago’s 343 neighborhoods. Children falling within 7 age cohorts, including infancy (mean age, 6 months) and ages 3, 6, 9, 12, 15, and 18 years, were sampled from randomly selected households and studied over 3 waves of data collection, to the early 2000s.^14^ Modal birth years of the original age cohorts were 1978, 1981, 1984, 1987, 1990, 1993, and 1996.

In 2011 to 2012, the PHDCN was extended through a random sample of wave 3 participants. Resource constraints prohibited following-up with the entire sample. Therefore, a 60% random sample of 4 of the original 7 PHDCN cohorts (infancy and age cohorts of 9, 12, and 15 years) with high retention rates at wave 3 (78.6%) and a wide age range was selected, yielding 1057 participants at wave 4 (63% response rate).^15^

In 2021, wave 4 respondents were followed-up for a fifth survey wave no matter where they lived in the US, yielding a sample of 682 respondents (66% response rate). We examine 2418 Black, Hispanic, and White respondents from the 4 sampled cohorts, including 1770 original members who were not part of the subsample drawn at wave 4. The difference between the original sample and the analytic sample size reflects subsampling at wave 4 and attrition, which we address through weights, and the fact that our youngest cohort was not asked questions about exposure to firearm violence until they were adults in wave 5. Additional information on the study design has been previously published.^15^

The current analysis also draws on data from the Gun Violence Archive (GVA), a not-for-profit corporation that aggregates data on incidents of gun violence from more than 7500 sources, including police departments, media, and government. We obtained all incidents throughout the US that occurred within 250 m of the respondents’ addresses in the year prior to respondents’ wave 5 interview dates in 2021.

**Figure 1.**
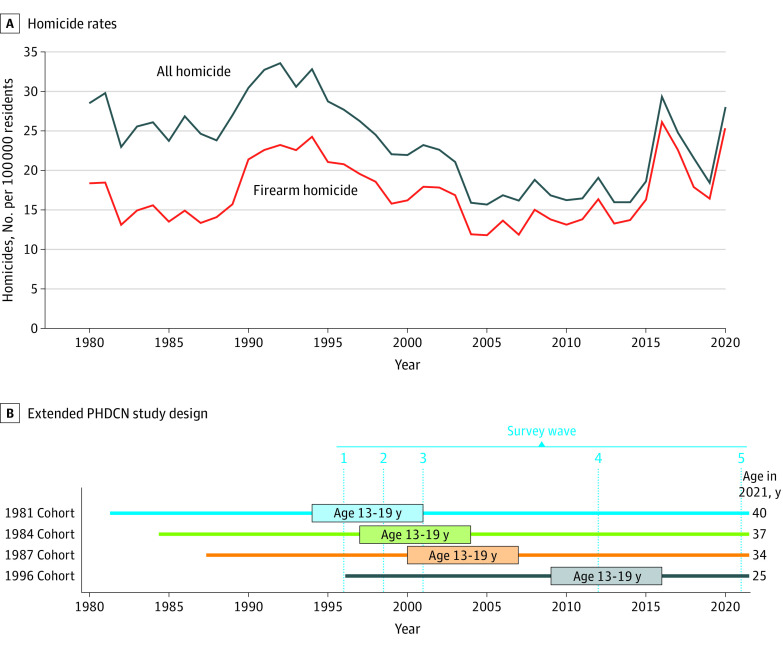
Homicide Rates in Chicago, Illinois, and the Extended Project on Human Development in Chicago Neighborhoods (PHDCN) Study Design A, Homicide rates were calculated using counts of homicide victims from Chicago Police Department records and interpolated population counts for the City of Chicago from the US Census Bureau decennial census and American Community Survey. B, Boxes highlight when members of each age cohort were aged 13 through 19 years, considered high-risk ages for participation in and exposure to gun violence. Dotted blue lines indicate midpoints of rolling survey data collection for each wave.

To illustrate the historical context of the data collection, we plot the ages of respondents and homicide rates in Chicago in Figure 1. The wave 1 survey in the mid-1990s coincided with the beginning of a long decline in lethal violence in Chicago, as in the rest of the country.^6,10^ Our oldest age cohort of respondents, born in 1981, reached their teenage years (ie, age 13-19 years) in the early 1990s when lethal firearm violence was at its peak. In contrast, the 1987 cohort, born only 6 years later, reached the formative years of adolescence in a much safer context. The 1984 cohort came of age between these periods, when crime was still high but declining. The youngest cohort, born in 1996, reached their teenage years at the lowest levels of lethal violence in both the US and Chicago in more than 40 years. However, by the time the 1996 cohort reached late adolescence and early adulthood, beginning in 2015 to 2016, Chicago was experiencing a surge in violence. By their 20th birthdays, these individuals were confronted by a dramatically more violent context than members of the other cohorts at age 20 years, despite being born into a period of rapidly declining violence. These patterns motivate our substantive focus on estimating cohort differences, in addition to race and sex inequalities, in exposure to firearm violence over the life course during these changing times.

### Measures and Variables

Age when first saw someone shot (ie, seen shot) was calculated from questions in waves 1, 2, 3, and 5. Respondents were asked if they had ever seen or been present when someone was shot, when this last occurred, if they had seen someone shot in the past year, and at what age they had first seen this. Respondents were not asked about the age of exposure in wave 5. The combination of these responses results in either a year of age or interval of ages in which first exposure occurred (eAppendix 1 in Supplement 1). Age when first shot (ie, been shot) was calculated from questions in waves 2, 3, and 5 asking respondents if they had ever been shot, the age this first occurred, and if they had been shot in the past year. Nearby shootings is the count of shootings resulting in a death or injury recorded by GVA that occurred in the past year within 250 m of a respondent’s residential location as reported at wave 5. This radius was chosen to capture events happening within the approximate distance of a standard Chicago city block (results are similar using distances of 100, 500, or 1000 m).

Race of respondents was reported by their primary caregiver, which was categorized first by ethnicity (Hispanic or non-Hispanic) and then by racial group (Black or White) for those who were non-Hispanic. We excluded 102 individuals (4.0%) of other races (eg, American Indian, Asian, or Pacific Islander) because the sample was too small to conduct longitudinal analyses. Sex was recorded by interviewers. Cohort was defined by age at enrollment in the study. Respondents were sampled by eligible ages (ie, infancy and ages 9, 12, and 15 years at wave 1) during the first wave of data collection, which occurred over approximately 3 years. Because of this design, there is variation in birth year within age cohorts. To ease interpretation of results, we refer to cohorts by their modal birth year (ie, 1981, 1984, 1987, and 1996).

### Statistical Analysis

We estimated survival curves of the cumulative percentage of respondents exposed to firearm violence by race, sex, and cohort by a given age using the nonparametric Turnbull maximum-likelihood estimator (NPMLE), a generalization of the Kaplan-Meier estimator applicable to interval-censored data.^16,17^ Accounting for interval censoring is necessary for the seen shot outcome because age at time of exposure is uncertain for respondents exposed in the years between wave 3 and 1 year prior to wave 5. This is less consequential for the been shot outcome, since wave 5 respondents were asked for the age when first shot, resulting in smaller censoring intervals.

We estimated associations between sociodemographic characteristics and exposure to firearm violence using multivariable semiparametric Turnbull (SPT) proportional hazards models.^18,19^ The SPT model is analogous to the Cox proportional hazards model but estimates the baseline hazard using the NPMLE to account for interval censoring. A Cox model with interval midpoints as exposure times yields similar estimates (eFigure 1 in Supplement 1). No formal test of the proportional hazards’ assumption exists for SPT models, but accelerated failure time models that relax the proportional odds assumption produce substantively equivalent estimates (eFigure 2 and eAppendix 2 in Supplement 1).

We used a negative binomial regression model to estimate the association between our covariates and counts of past-year shootings occurring within 250 m of a respondent’s residence. These models include combined survey design and attrition weights to permit inferences to the population of Chicago children. We performed statistical analyses in R software version 4.2.2 using the interval, icenReg, and MASS packages (R Project for Statistical Computing), with a 2-tailed significance level of *P* < .05.^17,19,20^ Because our analyses are descriptive and not directed at formal hypothesis testing, the purpose of calculating CIs is to draw inferences from our sample to the population. Data analyses were conducted from May 2022 to March 2023.

## Results

The overall sample size was 2418 respondents, with 1209 males (50.00%) and 1209 females (50.00%) and 890 Black respondents, 1146 Hispanic respondents, and 382 White respondents. By age 40 years, 6.46% of respondents had been shot and 50.00% of respondents had seen someone shot. Seeing others shot tended to occur earlier in life (mean [SD] age of exposure, 14.26 [5.83] years) than being shot (mean [SD] age of exposure, 17.11 [6.97] years). Counts of recent shootings within 250 m of a respondent’s residence were concentrated among a small number of sample members (mean [SD], 0.45 [2.04] shootings; maximum, 15 shootings).

**Figure 2.**
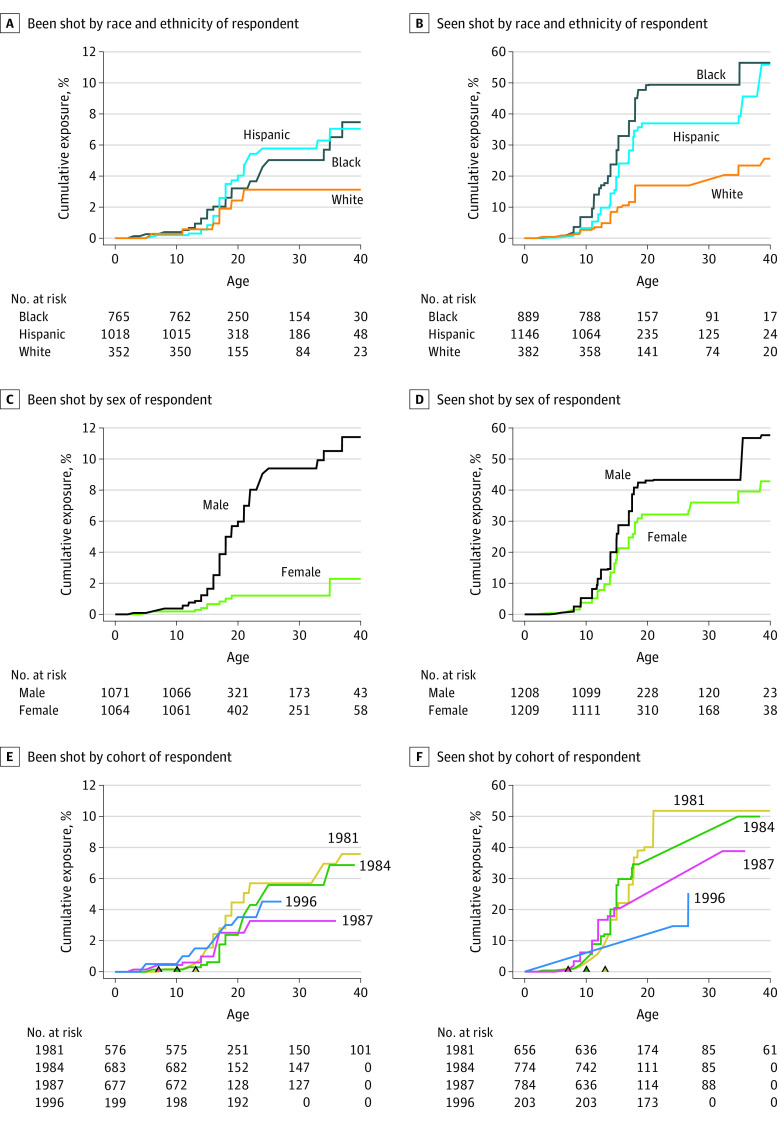
Cumulative Exposure to Gun Violence to Age 40 Years Survival curves were estimated using the Turnbull nonparametric maximum likelihood estimator for interval-censored events. Interval censoring results in plateaus in curves between survey waves 3 and 5 and regions where curves are not uniquely identified, indicated by diagonal lines. The observed data are consistent with any increasing line connecting the ends of the diagonal segments. At-risk respondent counts are model estimates as the exact number of respondents at-risk at any time is unknown due to interval censoring. Initial at-risk respondents are different between seen shot (2417 respondents) and been shot (2135 respondents) outcomes because been shot questions were not asked in the first survey wave. Triangles at base of panels E and F indicate the approximate age of each cohort at the time of the 1994 violence peak in Chicago (see Figure 1).

Figure 2 presents cumulative exposure curves by race, sex, and cohort. Racial differences in exposure to firearm violence were pronounced, with both forms of exposure lowest for White respondents (Figure 2A). By age 40, 7.47% of Black respondents and 7.05% of Hispanic respondents had been shot, with 1 Black respondent and 1 Hispanic respondent having been fatally shot. In contrast, 3.13% of White respondents had been shot by age 40 years. Racial and ethnic differences were also strongly age-patterned: no White respondents but 7 Black respondents and 5 Hispanic respondents were shot after age 21 years. Similarly, 56.34% of Black respondents and 55.75% of Hispanic respondents saw someone shot by age 40 years, compared with 25.53% of White respondents (Figure 2B).

Sex differences in being shot were also significant, with the cumulative exposure of male respondents (11.40%) 5 times higher than that of female respondents (2.28%) by age 40 years (Figure 2C). Differences again were age-patterned. Most reports of being shot for female respondents occurred during their midteenaged years, whereas males reported being shot throughout their teenaged years and their 20s and 30s. Sex differences in seeing others shot by age 40 years were more modest: 42.81% of female respondents and 57.64% of male respondents had seen someone shot (Figure 2D).

Direct experience of being shot was typically highest at all ages for the 1981 cohort, whose adolescence coincided with the 1990s peak in violence (Figure 2E). All cohorts displayed rapid increases in incidence of experiencing gun violence in late adolescence, except for the 1996 cohort, whose increase began earlier and was more gradual. The cumulative incidence of experiencing gun violence by the end of follow-up for the 1996 cohort (ie, in their mid-20s) fell between the high-exposure cohorts (1981 and 1984) and low-exposure cohort (1987).

Similar to direct experience of being shot, the cumulative risk of having seen someone shot was higher for the 1981 and 1984 cohorts (Figure 2F). The 1987 cohort reached mid-to-late adolescence during the low-violence period of the early 2000s, whereas the 2 older cohorts reached the same developmental stage in a more violent era in the 1990s, with greater exposure among the latter 2 cohorts, despite the 1987 cohort initially having had more exposure in early childhood. The 1996 cohort experienced lower exposure than the other cohorts. At the end of the observation period, at approximately ages 26 to 27 years, the cumulative risk of having seen someone shot for the 1996 cohort was approximately the same as when the older cohorts were just age 15 years.

**Figure 3.**
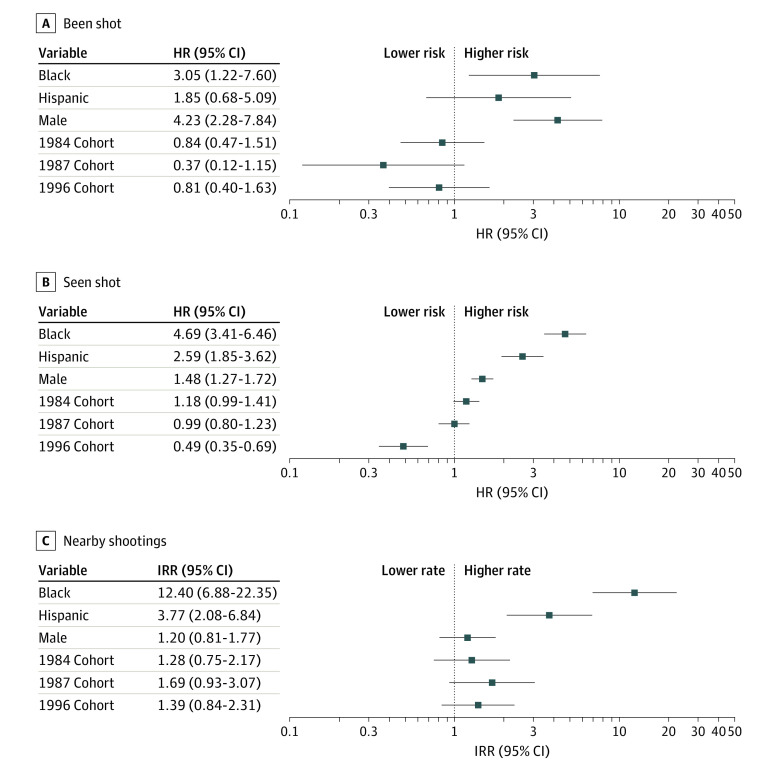
Model Estimates of Inequalities in Exposure to Gun Violence Hazard ratios (HR) and incidence rate ratios (IRR) greater than 1 indicate higher estimated exposure to gun violence. HRs were estimated with multivariable semiparametric Turnbull proportional hazards models. IRRs were estimated with a multivariable negative binomial regression. Reference categories are White for race, female for sex, and 1981 for cohort. Models were weighted to permit generalizing estimates of exposure to the population of children growing up in Chicago in the 1990s. The case weights applied during estimation are the product of the survey design weights and attrition weights at each wave, constructed using estimated probabilities from binary logistic regression models.^15^ Observations received the combined weight at their final follow-up period (eg, an individual observed through wave 3 would receive a weight equal to the product of the survey design weight, their wave 2 attrition weight, and their wave 3 attrition weight). Results are insensitive to trimming weights to the center 90% of the distribution. Results are also similar with alternative specifications of survival models (eFigure 2 in Supplement 1) and without weights (eFigure 3 in Supplement 1). Sample sizes are 2417 respondents for seen shot, 2135 respondents for been shot, and 649 respondents for nearby shootings.

Figure 3 presents adjusted hazard ratios (aHRs) and adjusted incidence rate ratios (aIRRs) with 95% CIs from multivariate SPT models of been shot and seen shot, as well as the negative binomial model of nearby shootings. Independent measures of nearby shootings provide a complement to self-reported indicators of being shot and seeing someone shot. We assess proximity to shootings by race, sex, and cohort during a period of elevated violence in 2020 to 2021.

Statistical power is low for estimates of been shot due to the rarity of the event (Figure 3A). Hence, 95% CIs are wide. The hazard of being shot was more than 3 times as high for Black individuals (aHR, 3.05; 95% CI, 1.22-7.60) compared with White individuals. The difference between Hispanic and White individuals was not significant (aHR, 1.85; 95% CI, 0.68-5.09). Compared with female respondents, hazards of being shot were more than 4 times higher for male respondents (aHR, 4.23; 95% CI, 2.28-7.84). Although the 1987 cohort exhibited the lowest hazard of being shot (aHR, 0.37; 95% CI, 0.12-1.15) compared with the 1981 cohort, which exhibited the highest estimated exposure, the difference between the cohorts was not statistically significant (Figure 2E). The 1996 and 1984 cohorts exhibited hazards similar to the 1981 cohort but these differences were also not significant (1996: aHR, 0.81; 95% CI, 0.40-1.63; 1984: aHR, 0.84; 95% CI, 0.47-1.51).

Analyses of the hazard of witnessing violence found that, compared with White individuals, the hazard of seeing someone shot was more than 4.5 times as high for Black individuals (aHR, 4.69; 95% CI, 3.41-6.46) and more than 2.5 times as high for Hispanic individuals (aHR, 2.59; 95% CI, 1.85-3.62) (Figure 3B). Compared with female respondents, male respondents were significantly more likely to have seen someone shot (aHR, 1.48; 95% CI, 1.27-1.72). Finally, consistent with results presented in Figure 2F, SPT results indicate that the 3 older cohorts had approximately similar hazards of seeing someone shot, but the 1996 cohort exhibited a hazard approximately half that of the 1981 cohort (aHR, 0.49; 95% CI, 0.35-0.69).

Figure 3C presents estimates of the correlates of the count of shootings within 250 m of a respondent’s residence during the 12 months preceding their wave 5 interview. While there were no significant differences by sex or cohort in proximity to nearby shootings, we observed significant racial disparities. Black individuals (aIRR, 12.40; 95% CI, 6.88-22.35) and Hispanic individuals (aIRR, 3.77; 95% CI, 2.08-6.84) experienced higher rates of shootings in close proximity to their residences than White individuals. Notably, we detected no race or sex by cohort interactions for this measure or for having been shot or having seen someone shot.

**Figure 4.**
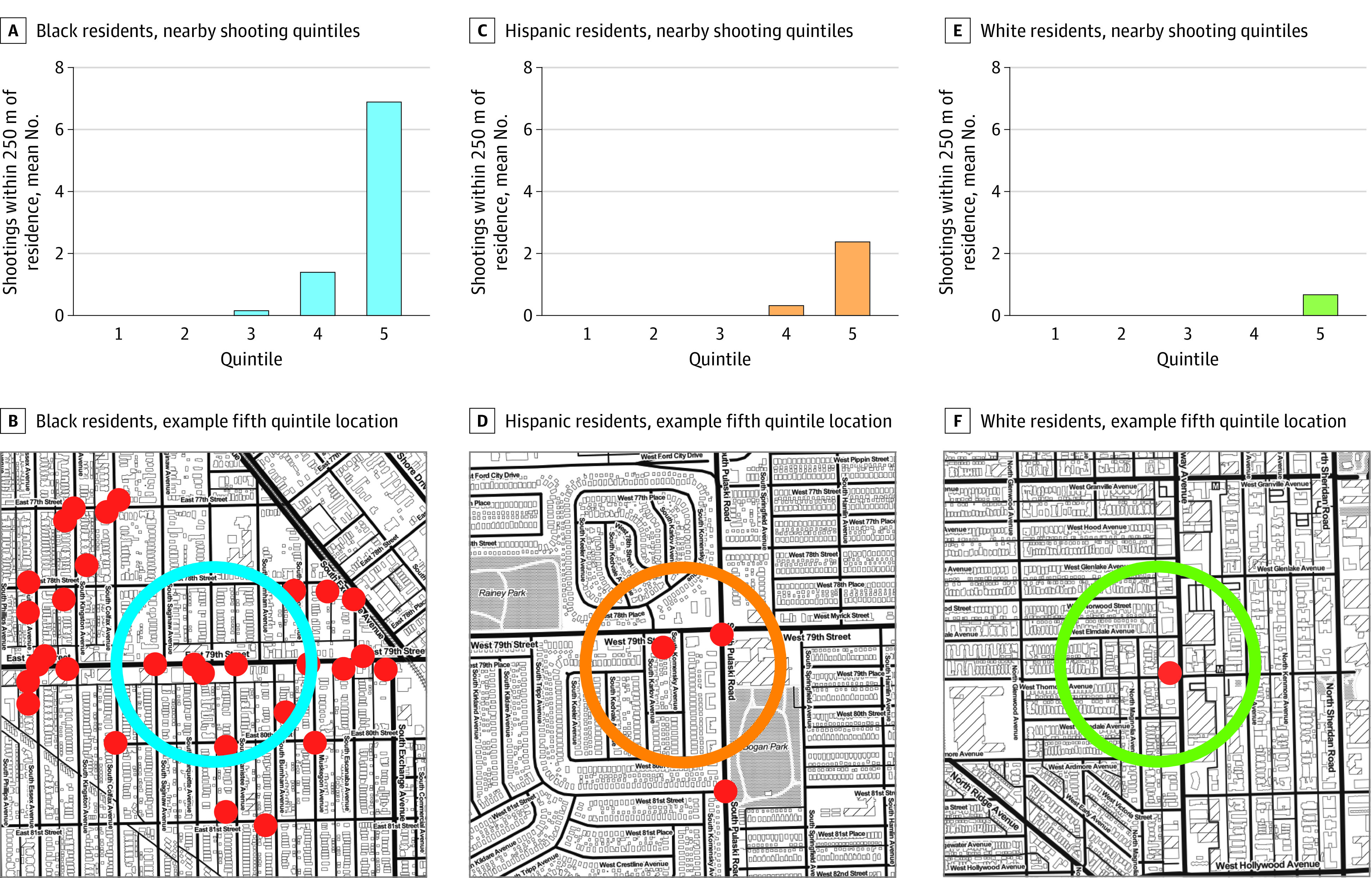
Frequency of Nearby Shootings in the Past Year by Respondent Race A, C, and E depict race-specific quintiles of frequencies of shootings within 250 m of respondents' residence in the past year. B, D, and F depict representative locations with counts of nearby shootings approximately equal to the mean of each group's fifth quintile (7 shootings for Black respondents, 2 shootings for Hispanic respondents, and 1 shooting for White respondents). Red dots are shootings. Circles are 250-m radii around respondent places of residence but are shifted to protect anonymity. Only race differences are shown because nearby shooting frequencies did not notably differ by sex or age cohort. Analysis was restricted to Black, Hispanic, and White respondents of wave 5 (649 respondents). Map tiles by Stamen Design; map data by OpenStreetMap (OpenStreetMap Foundation).

Figure 4 depicts the magnitude of these race differences in terms of raw counts. In the most violent neighborhoods (ie, the fifth quintile, denoting the top 20% of residential locations by the frequency of shootings), a mean of 7 shootings occurred in the preceding year within 250 m of the residences of Black respondents, compared with more than 2 for Hispanic respondents and less than 1 for White respondents.

## Discussion

In this cohort study, the accelerated longitudinal design of the extended PHDCN allowed us to examine multiple forms of firearm violence exposure from childhood through midadulthood in a population sample. We found significant differences in exposure to firearm violence by race and sex, and our findings on cohort differences point to changing societal conditions as key factors associated with whether and at what life-stage individuals were exposed to firearm violence. For instance, participants from the 1987 cohort had the lowest incidence of having been shot, which could have been because they reached the formative years of late adolescence when firearm violence was at its lowest point in the past 3 decades. Examples of other changing conditions that merit future research include the surge in firearm purchasing in the wake of the COVID-19 pandemic, the loosening of gun regulations in the US, and widespread unrest over excessive police use of force in Black communities.

Recent surges in lethal firearm violence have captivated public and media attention, but the reach of firearm violence is broader than immediate fatalities. Particularly for Black and Hispanic males growing up in urban neighborhoods, seeing someone shot or being shot before even reaching age 20 years is pervasive. And for Black and Hispanic individuals who reached adulthood without being exposed to firearm violence, there was still a considerable likelihood that they would be exposed by age 40 years. In addition, at the time of our most recent survey in 2021, Black and Hispanic respondents were residing in communities with firearm violence that far exceeded the rates in the communities of White respondents.

### Implications for Public Health

A broader focus beyond fatalities to include nonfatal gunshot injuries and witnessing of incidents is critical for understanding the full health outcomes associated with firearm violence. In addition, the sustained stress resulting from routine exposure to firearm violence can take a cumulative physiological toll on the body, and is associated with damage to the body’s regulatory system and the acceleration of aging and susceptibility to disease.^21,22,23^ To the extent that major and potentially chronic stressors are disproportionately endured by some sociodemographic groups, as we have shown here, variation in these stressors may contribute to dramatic sociodemographic differences in health.^24^ The stress from chronic exposure to firearm violence may also contribute to subsequent violence, through its impact on aggression, or even through the normalization of violence.^25,26^

### Future Research

Consistent with the life-course framework of the current study, future work should focus not simply on the consequences associated with having ever seen someone shot or having been shot themselves, but also the timing of the exposure and accumulation of exposures. Furthermore, a focus on the consequences of exposure to firearm violence should examine both near-term and long-term outcomes. Additionally, further research is necessary to explain the variations in firearm exposure by race, sex, and cohort that were identified in this study, including studies that examine the role of residential segregation, as well as residential mobility.

### Limitations

This study has some limitations. Although this long-term study uses a representative sample of children who reached the formative years of adolescence at differing times over the last quarter-century, it is based on children originally from Chicago. This raises generalizability questions, but violence rates and trends in Chicago parallel those in other major cities in the US, such as Philadelphia, Pennsylvania. Furthermore, the extended PHDCN data collection followed-up with respondents wherever they moved. Although retention rates compare well with contemporary urban samples and the analyses were weighted to reflect the sampling design and attrition, selection out of the sample on unobserved covariates may bias the results. Another potential limitation, common to self-report surveys, is recall error, such as difficulty in recalling the precise timing of events.

A 2020 study^27^ using GVA data found undercounting of nonfatal shootings due to selective reporting of firearm violence by the media. However, race or ethnicity of individuals who were shot was not associated with the likelihood of reporting, suggesting the racial and ethnic differences in nearby shootings reported in this study are unlikely to be biased by selective reporting. Moreover, we found similar racial and ethnic differences when restricting our analyses to fatal shootings.

## Conclusions

This cohort study reports on previously undocumented inequalities in exposure to firearm violence over the life course by race, sex, and cohort. Life-course exposure to firearm violence was significantly and persistently higher for Black and Hispanic individuals. Sex disparities in witnessing and proximity to firearm violence were small, but men were at significantly higher risk of being shot than women. Cohort differences were most pronounced for having seen someone shot; in particular, the youngest cohort in our study had a lower likelihood of having seen someone shot than counterparts born in earlier decades. The perspective we have taken reorients the study of exposure to firearm violence toward a focus on sex and racial inequalities over the life course in the context of ongoing social changes that differentiate the life experience of successive birth cohorts.

## Notes

Supplement 1. **eAppendix 1.** Survey Measures **eFigure 1.** Cox Proportional Hazard Model Estimates **eAppendix 2.** Accelerated Failure Time Models **eFigure 2.** Interval Censored Accelerated Failure Time Models **eFigure 3.** Unweighted Model Estimates

Supplement 2.
Data Sharing Statement
