# Supplementary material for: Inequalities in Exposure to Firearm Violence by Race, Sex, and Birth Cohort From Childhood to Age 40 Years, 1995-2021
Source: JAMA Netw Open. 2023 May 9;6(5):e2312465. doi: 10.1001/jamanetworkopen.2023.12465 (PMC10170342; doi:10.1001/jamanetworkopen.2023.12465)
Supplement: Supplement 1. — eAppendix 1. Survey Measures eFigure 1. Cox Proportional Hazard Model Estimates eAppendix 2. Accelerated Failure Time Models eFigure 2. Interval Censored Accelerated Failure Time Models eFigure 3. Unweighted Model Estimates [file jamanetwopen-e2312465-s001.pdf]

Supplemental Online Content

Lanfear CC, Bucci R, Kirk DS, Sampson RJ. Inequalities in exposure to firearm violence by race, sex, and birth cohort from childhood to age 40 years, 1995-2021. *JAMA Netw Open*. 2023;6(5):e2312465. doi:10.1001/jamanetworkopen.2023.12465

eAppendix 1. Survey Measures
eFigure 1. Cox Proportional Hazard Model Estimates
eAppendix 2. Accelerated Failure Time Models
eFigure 2. Interval Censored Accelerated Failure Time Models
eFigure 3. Unweighted Model Estimates

This supplemental material has been provided by the authors to give readers additional information about their work.

© 2023 Lanfear CC et al. *JAMA Network Open.*

eAppendix 1. Survey Measures

The following survey questions were used to create age-specific measures of exposure to firearm violence and personal firearm victimization.

Age when first witnessed someone being shot (“seen shot”) was calculated from several questions across the study period.

- Wave 1 (1981, 1984, and 1987 cohorts)
  - “Have you ever seen or been present when someone was shot?”
  - “When was the last time you saw that?”
- Wave 2 (1981, 1984, and 1987 cohorts)
  - “In your whole life, have you ever seen someone else get shot?”
  - “Now just thinking about the last 12 months, have you seen someone get shot?”
  - “How old were you the first time you saw this?”
- Wave 3 (1981, 1984, and 1987 cohorts)
  - “In your whole life, have you ever seen someone else get shot?”
  - “Now just thinking about the last 12 months, have you seen someone get shot?”
- Wave 5 (1981, 1984, 1987, and 1996 cohorts)
  - “In your whole life, have you ever seen someone else get shot?”
  - “Now just thinking about the past 12 months, have you ever seen someone else get shot?”

Age at first personal gunshot victimization (“been shot”) was calculated in a similar manner, relying on questions at Waves 2, 3 and 5.

- Wave 2 (1981, 1984, and 1987 cohorts)
  - “In your whole life, have you ever been shot?”
  - “Now just thinking about the last 12 months, have you been shot?”
- Wave 3 (1981, 1984, and 1987 cohorts)
  - “In your whole life, have you ever been shot?”
  - “About how old were you the first time this happened?”
  - “Now just thinking about the last 12 months, have you been shot?”
- Wave 5 (1981, 1984, 1987, and 1996 cohorts)
  - “In your whole life, have you ever been shot?”
  - “How old were you when this first happened?”

No survey questions regarding exposure to firearm violence and victimization were asked in the wave 4 survey.

© 2023 Lanfear CC et al. *JAMA Network Open.*

eFigure 1. Cox Proportional Hazard Model Estimates

A Been shot

Variable	HR (95% CI)
Black	3.02 (0.99–9.27)
Hispanic	1.84 (0.60–5.58)
Male	4.18 (1.88–9.29)
1984 Cohort	0.80 (0.31–2.07)
1987 Cohort	0.35 (0.10–1.17)
1996 Cohort	0.82 (0.30–2.19)

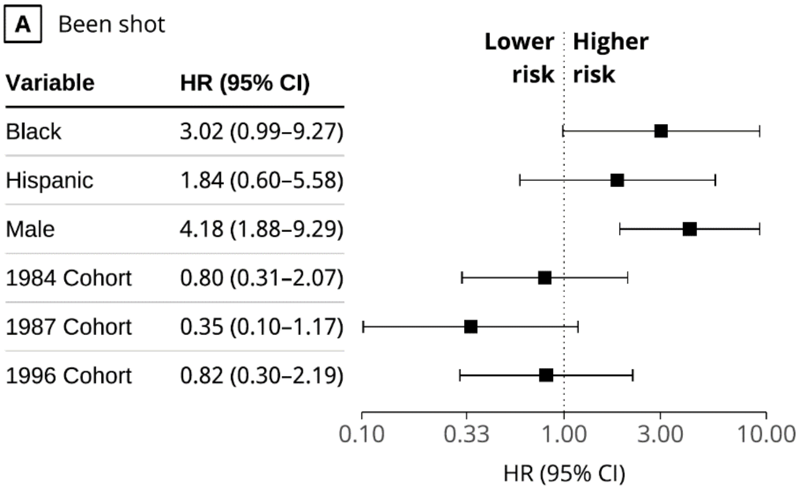

B Seen shot

Variable	HR (95% CI)
Black	4.70 (3.24–6.81)
Hispanic	2.57 (1.78–3.72)
Male	1.48 (1.20–1.82)
1984 Cohort	0.96 (0.76–1.21)
1987 Cohort	0.69 (0.52–0.91)
1996 Cohort	0.53 (0.31–0.91)

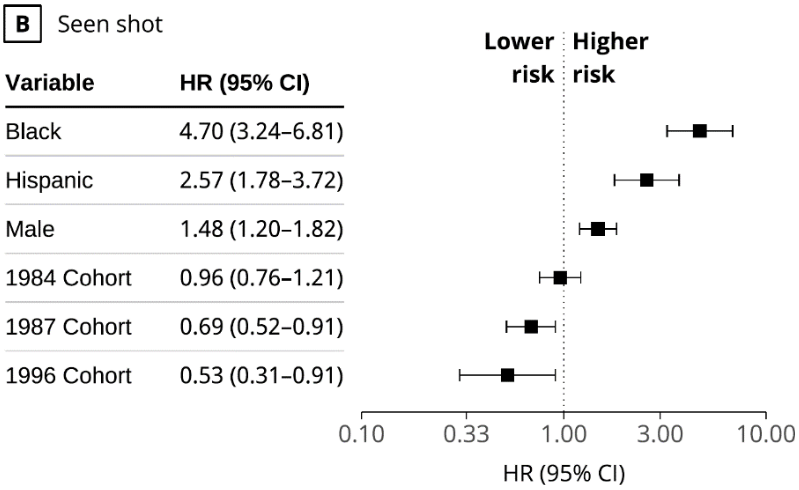

Hazard ratio (HR) estimates from multivariable Cox proportional hazards models are provided here for comparison to the main text estimates from multivariable SPT proportional hazards models. Cox proportional hazards models cannot accommodate interval censored data. Here event times are assumed to occur at censoring interval midpoints to permit use of the Cox model. Models were fit using the same combined weights for survey design and attrition as in the main text. Reference categories are White for race, Female for sex, and 1981 for cohort.

© 2023 Lanfear CC et al. *JAMA Network Open.*

eAppendix 2. Accelerated Failure Time Models

The SPT models in the main text and Cox models above make a proportional hazards assumption—covariates influence survival by proportionally increasing or decreasing a common age-specific baseline hazard. When the proportional hazards assumption is violated, estimated hazard ratios will be biased. Additionally, SPT and Cox models estimate the baseline hazard non-parametrically, permitting it to conform closely to the data and avoiding bias that may result from mis-specified parametric baseline hazards. In contrast, accelerated failure time (AFT) models assume covariates influence survival by multiplicatively accelerating or decelerating a parametrically-specified survival function. That is, rather than assuming covariates scale a non-parametric baseline hazard upward and downward, covariates multiplicatively increase or decrease the rate of progression through a common parametric baseline survival curve. Due to the sensitivity of AFT models to the choice of parametric specification, proportional hazard models are typically considered the default approach for modeling time-to-event data, so long as the proportional hazards assumption appears warranted. In the present case, interval censoring prevents formal tests of the proportional hazards assumption, but non-parametric (i.e., NPMLE) survival curves (figure 2) and theoretical expectations provide guidance.

Theoretically, we expect the proportional hazards assumption to be more tenable for race and sex covariates: these will increase or decrease the hazard of exposure, which generally follows a shared non-monotonic, unimodal hazard curve that emerges as a result of the age crime curve and changes in routine activities over the life course. That is, it seems reasonable to expect members of some demographic groups to have a fixed multiplicatively higher likelihood of seeing shootings or being shot at any given age. Evidence in support of this is provided by the survival curves in figure 2, panels A–D which generally appear to shift proportionally across sex and race.

We expect the proportional hazards assumption to be less tenable for the cohort differences. For the oldest three cohorts, firearm homicide in Chicago was at its highest from 1990 to 1995 and then precipitously declined for ten years. As a result, the 1981 cohort experienced a context of high-violence well into adolescence while violence had declined greatly by the time the 1987 cohort entered adolescence. The 1996 cohort, in contrast, lived in a low violence context until the end of adolescence. As a result, each cohort's period of peak violence occurred at a different age. We would expect this results in both a proportional hazards shift—due to differences in overall level of violence—and an acceleration of failure time—as risk of exposure is highest during peak periods. In support of the latter expectation, the survival curves in figure 2, panels E and F, share a similar shape shifted forward and backward between cohorts. The highest expected exposure should have occurred when peak environmental risk overlapped with high age-specific risk (i.e., for the 1981 cohort), though the models cannot account for both simultaneously while addressing interval censoring.

AFT models may be more appropriate for modeling cohort differences as they relax the proportional hazards assumption and instead model acceleration of failure time. However, AFT estimates may be biased if the baseline hazard curve is misspecified. eFigure 2 depicts results from five common AFT baseline parameterizations. Estimates were similar across all specifications and to the proportional hazards models—except for cohorts, where differences are larger in AFT models. The log-normal specification produces the best AIC value in all cases except for been shot with survey weights where the log-logistic parameterization is slightly preferred (though similar to Weibull, gamma, and generalized gamma). Models using the highly flexible three parameter generalized gamma distribution—which makes strong demands of the data—failed to converge for the seen shot outcome. In all cases the results from AFT models closely parallel those from the main-text SPT models in direction, relative magnitude, and statistical significance (see figure 2), both with and without survey weights (see eFigure 3, panels A and B).

© 2023 Lanfear CC et al. *JAMA Network Open.*

eFigure 2. Interval Censored Accelerated Failure Time Models

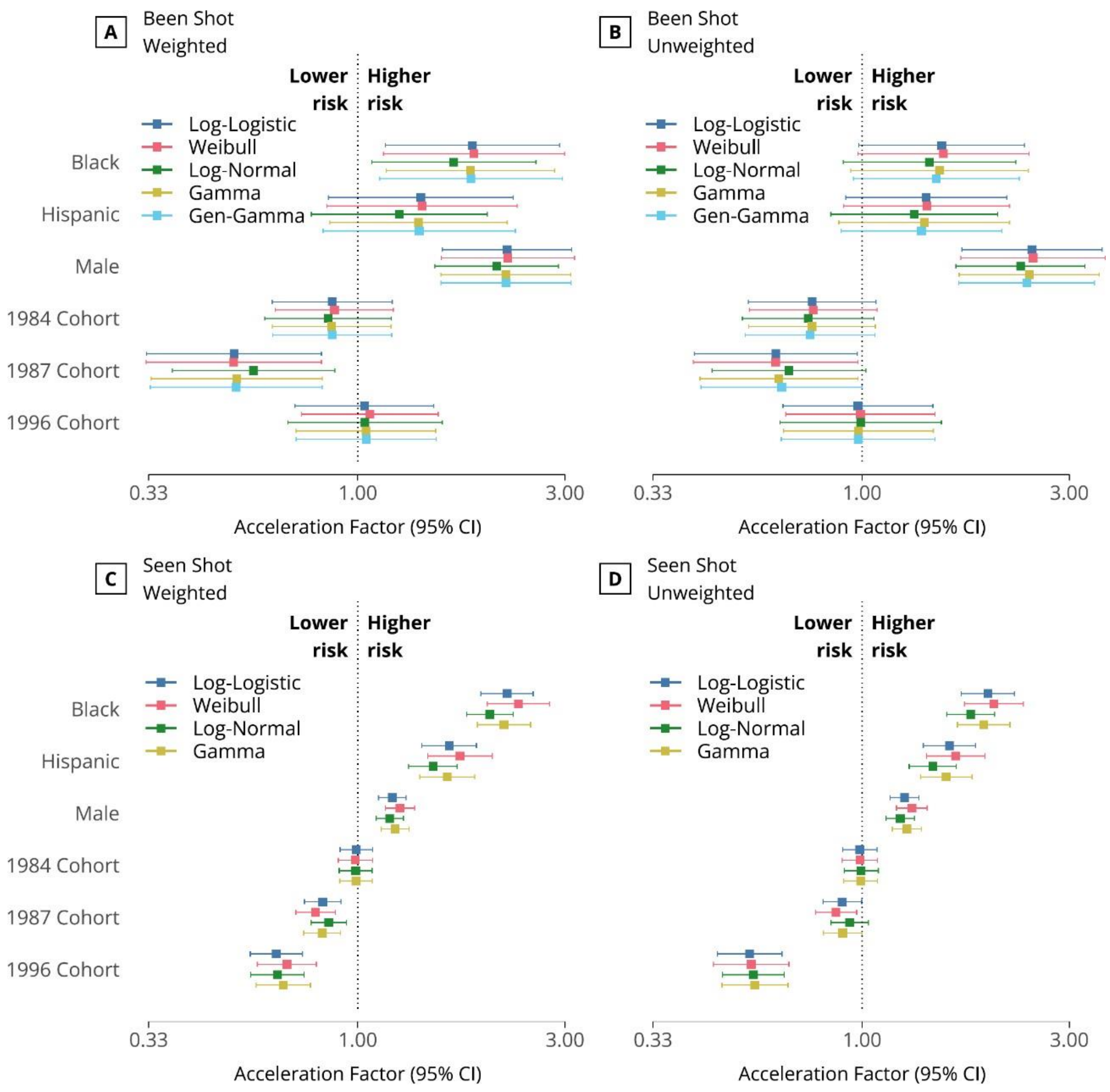

Squares represent acceleration factor point estimates from AFT models. AFT models relax the proportional hazards assumption of the main text SPT proportional hazards models but are sensitive to the distribution chosen, so five common parametric specifications are presented. Results are similar across all specifications but the best fit by AIC is obtained from the log-normal model in all panels except A, where the log-logistic specification is preferred. The generalized gamma distribution, which makes strong demands of the data, is omitted for seen shot because it failed to converge. Reference categories are White for race, Female for sex, and 1981 for cohort.

© 2023 Lanfear CC et al. *JAMA Network Open.*

eFigure 3. Unweighted Model Estimates

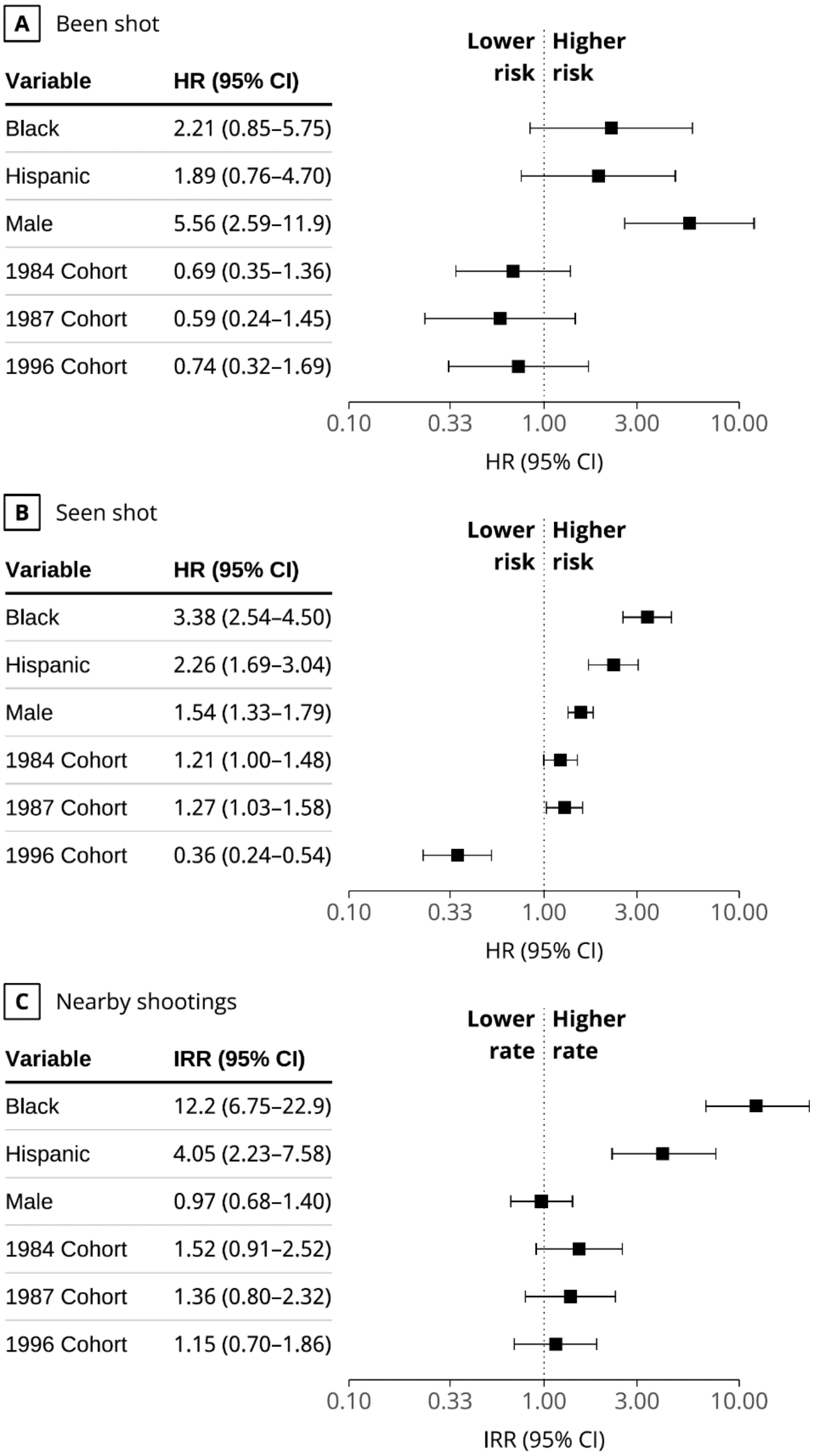

A Been shot

Variable	HR (95% CI)
Black	2.21 (0.85–5.75)
Hispanic	1.89 (0.76–4.70)
Male	5.56 (2.59–11.9)
1984 Cohort	0.69 (0.35–1.36)
1987 Cohort	0.59 (0.24–1.45)
1996 Cohort	0.74 (0.32–1.69)

B Seen shot

Variable	HR (95% CI)
Black	3.38 (2.54–4.50)
Hispanic	2.26 (1.69–3.04)
Male	1.54 (1.33–1.79)
1984 Cohort	1.21 (1.00–1.48)
1987 Cohort	1.27 (1.03–1.58)
1996 Cohort	0.36 (0.24–0.54)

C Nearby shootings

Variable	IRR (95% CI)
Black	12.2 (6.75–22.9)
Hispanic	4.05 (2.23–7.58)
Male	0.97 (0.68–1.40)
1984 Cohort	1.52 (0.91–2.52)
1987 Cohort	1.36 (0.80–2.32)
1996 Cohort	1.15 (0.70–1.86)

Main text models were fit using combined weights for survey design and attrition. This figure presents results from the same models estimated without weights for comparison. Hazard ratios (HR) and incidence rate ratios (IRR) greater than 1 indicate higher estimated exposure to firearm violence. HRs were estimated with a SPT proportional hazards model. IRRs were estimated with a negative binomial regression. Reference categories are White for race, Female for sex, and 1981 for cohort.

© 2023 Lanfear CC et al. *JAMA Network Open.*
