# Supplementary material for: Inequalities in Exposure to Firearm Violence by Race, Sex, and Birth Cohort From Childhood to Age 40 Years, 1995-2021
Source: JAMA Netw Open. 2023 May 9;6(5):e2312465. doi: 10.1001/jamanetworkopen.2023.12465 (PMC10170342; doi:10.1001/jamanetworkopen.2023.12465)
Supplement: Supplement 2. — Data Sharing Statement [file jamanetwopen-e2312465-s002.pdf]

Data Sharing Statement

Lanfear. Inequalities in Exposure to Firearm Violence by Race, Sex, and Birth Cohort From Childhood to Age 40 Years, 1995-2021. *JAMA Netw Open*. Published May 09, 2023. doi:10.1001/jamanetworkopen.2023.12465

Data
Data available: Yes
Data types: Deidentified participant data
How to access data: Replication data and code will be made available upon publication and signed confidentiality agreement at https://dataverse.harvard.edu/. See https://doi.org/10.7910/DVN/YWZL5K.
When available: With publication

Supporting Documents
Document types: Statistical/analytic code
How to access documents: https://dataverse.harvard.edu/. See https://doi.org/10.7910/DVN/YWZL5K.
When available: With publication

Additional Information
Who can access the data: Researchers who agree to data use conditions.
Types of analyses: Replication
Mechanisms of data availability: Signed data agreement
